# Enhancing efficiency in a cardiac investigations department by increasing remote patient monitoring

**DOI:** 10.1093/intqhc/mzz065

**Published:** 2019-12-22

**Authors:** Paul Ryan, Caitriona McGrath, Iain Lawrie, Caoimhe Fitzsimons, Jack O’Shea, Aoife De BrÚn

**Affiliations:** 1 Cardiac Investigations, Mater Misericordiae University Hospital, Eccles St., Dublin 7, Ireland; 2 St. Luke’s Hospital, Rathfarnham, Dublin 6, Ireland; 3 Heart and Lung Transplant Centre, Mater Misericordiae University Hospital, Eccles St., Dublin 7, Ireland; 4 Emergency Department, Mater Misericordiae University Hospital, Eccles St., Dublin 7, Ireland; 5 Radiology Department, Mater Misercordiae University Hospital, Eccles St., Dublin 7, Ireland; 6 School of Nursing, Midwifery and Health Systems, University College Dublin, Dublin 4, Ireland

**Keywords:** remote monitoring, cardiology, quality improvement, patient safety

## Abstract

**Objective:**

Remote monitoring (RM) of patients with cardiac rhythm management devices enables healthcare teams to effectively and efficiently monitor patients with heart problems without the requirement in-person patient visits. RM has been associated with safer and higher quality care but was not being used to its full potential in this setting. Cardiac rhythm management had observed an average implant rate of 295 devices per year over the past 13 years, resulting in a five-fold growth in patient follow-up in clinics. This increased demand was becoming unmanageable, with impacts on care quality. This study aimed to enhance the enrolment of eligible patients to RM.

**Design:**

A pre-post design.

**Setting:**

A 600-bed city centre teaching hospital in Dublin, Ireland.

**Participants:**

Hospital staff and patients eligible for RM.

**Interventions:**

Lean Six Sigma methods were used to develop patient education materials on RM and the clinic area was redesigned to enable RM enrolment and monitoring.

**Main outcomes measures:**

Number of unscheduled attendances to clinic and RM enrolment.

**Results:**

At baseline, the clinic was processing 102 RM follow-up checks with 140 unscheduled attendances on average per month. Following implementation, RM enrolment increased to 335 RM follow-up checks (194% increase), with 41 unscheduled attendances on average per month (70% decrease). These results were sustained one-year post-implementation.

**Conclusions:**

These process changes have streamlined workflow by reducing the number of unscheduled attendances to clinic and increased the use of RM among the eligible patient population. This has meant safer, more timely responses to cardiac events and enhanced care quality.

## Introduction

Remote monitoring (RM) has long been the focus of healthcare providers involved in the implantation of cardiac rhythm management (CRM) devices. Modern technology now allows patients with heart problems and defects to continue with their lives as normally as possible by either transmitting data automatically from their implanted cardiac device (ICD) to a server from their homes to a cardiology device clinic or by allowing the patient to manually send data. This enables healthcare teams to effectively and efficiently monitor patients without the requirement for patients to visit clinics. This study employed the practices and principles of Lean Six Sigma (LSS) with the objective of designing a process to increase the number of patients availing of cardiac RM in order to allow for a more detailed monitoring of their heart health, decrease the number of in-person visits to cardiology device clinics, streamline workflow, and release time to educate patients on the benefits of RM in providing safer and higher quality care.

RM is a relatively new focus of healthcare and initially concerns were expressed over its cost effectiveness, safety and feasibility, particularly in terms of the potential to miss serious cardiac events. Despite being widely used, it was ten years after the introduction of this technology that the first randomized controlled trial on remote follow-up for patients with implanted cardiac devices was published. The TRUST study [1, 2] explored the impact of RM and the associated risks and benefits. The research demonstrated a reduction in follow-ups in patient’s physical attendance at clinics and also observed a significant reduction in the median response time for a detected arrhythmia from 3.5 days to 1 day [3]. This suggests that in fact RM enabled more rapid responses than traditional patient monitoring approaches. The authors did advise that since follow-up was evaluated over 15 months post-implantation, that it was unlikely to have identified the majority of device and lead problems. It was also found that in-hospital follow-up was associated with under reporting of device-related issues and a delay in the detection of clinical adverse events [1]. In contrast, RM has the capacity to deliver daily remote follow-up and has demonstrated enhanced discovery of both technical and clinical issues.

A series of randomized controlled trials exploring the impact and outcomes of RM have delivered some significant findings. The EFFECT study revealed the potential for reduced death associated with cardiovascular hospitalizations for patients with ICDs who were remotely monitored by a reduction in the rate of all-cause hospitalizations and a decreased average length of in-patient stay [4]. The In-Time Trial demonstrated a reduction in the number of inappropriate shocks (lead issues, e.g. failure or mis-reading of cardiac rhythm) in addition to reduced hospital stays for patients remotely monitored [5]. Two additional trials confirmed that RM reduced hospitalization and clinic visits [6, 7]. Finally, the CONNECT study showed both a reduction in the median time from the recording of a clinically actionable event to decision time and a decreased number of physical clinic visits [3]. Thus, it is evident from the literature that RM saves patient time in terms of excessive travel, reduces unnecessary visits to clinics, reduces number of inappropriate shocks, and allows earlier detection of arrhythmias and clinically actionable events [8]. Many scheduled visits do not require any intervention and are therefore both cost and resource consuming for a hospital, without benefit to the patient [9].

Ageing populations and advancements in cardiac treatments, including technological advancements in cardiac monitoring, have led to an increased burden on cardiac monitoring internationally [10]. This has led to calls to streamline services to optimize efficiency. The use of quality improvement methodologies in healthcare settings has become common in recent years as organizations struggle to maximize resources and reduce waste [11]. Although the evidence for LSS in healthcare is mixed [12], LSS has demonstrated some success in similar studies in the field of cardiology and work processes. For instance, Gijo *et al.* [13] used LSS methodology to reduce average outpatient waiting times from 57 to 24 minutes, Jackson [14] reduced wait time from 30 minutes to less than 15 minutes and Barrios *et al.* [15] reduced their waiting times since appointment request until patient care appointment from 6.89 days to 4.08 days. However, to our knowledge, LSS has not been applied to enhancing the use of remote monitoring of patients with implanted cardiac devices.

Given these trends and this increased burden on services, there was a local need to re-examine existing ways of working and to develop more appropriate processes that align with recent advancements in the field. This study adopted a novel approach to enhancing cardiac RM: employing Lean Six Sigma (LSS) methodology to re-design processes and enable the expansion of RM. Whilst the use and value of RM is well documented and the benefits to patient safety, morbidity and mortality are established [4, 5], this study also seeks to improve time management, work flow and optimize use of resources.

## Methods

### Study context

The setting for this research was a 600-bed city centre teaching hospital in the Mater Misericordiae University Hospital, Dublin, Ireland. As a national centre, it is responsible for a range of specialist services including cardiology and device management. In 2016, LSS quality improvement methodology was used to review work practices relating to outpatient services in the cardiac investigations department, specifically related to the RM of patients.

**Figure 1:**
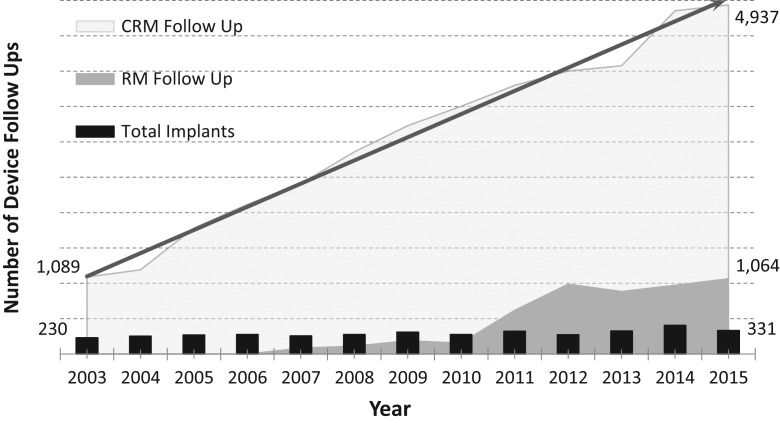
The increasing burden on cardiac remote monitoring.

Local data from the site of this study indicate that there has been a marked increase in the number of cardiac devices implanted, which in turn places significant burden on outpatient cardiology device clinics to deliver follow-up services. With an average implant rate of 295 devices a year over the last 13 years, and a typical follow-up time of 3–6 months (combined with a patient population living longer), the result has been a 133% growth in patient follow-up since 2006. The burden on these clinics continues to rise with patients requiring scheduled and unscheduled access. This increased demand reached a level that was almost unmanageable with follow-ups continuing to grow at unsustainable rates, impacting on quality of care with potential implications for patient safety (see Fig. 1).

### Project team

In 2016, a multidisciplinary project team was formed to assess the use of RM for cardiology patients. The team consisted of a cardiac physiologist, a clinical specialist radiation therapist, a clinical nurse facilitator, a radiology service manager and a clinical nurse specialist. Additionally, physiologists, nursing staff, medical staff, administration staff, IT staff, device manufacturers and patients were invited to participate at various points in the process.

According to the policy activities that constitute research at the Mater Misericordiae University Hospital, this work met criteria for operational improvement activities exempt from local ethics review. Approval for this project was granted by the Chief Executive Officer of the hospital.

### Design

The LSS DMAIC (Lean Six Sigma Design, Measure, Analyse, Improve and Control) approach was used to guide this project. LSS is a management philosophy originally developed in industry by Toyota (Lean) and Motorola (Six Sigma) that seeks to drive a quality culture change within an organization. The method attempts to identify and remove non-value added activities, often described as ‘waste’, while increasing the quality of the service delivered to the patient. Such waste is described as that which does not add value to a process, of which there are seven types: time, inventory, motion, waiting, over-processing, over-production and defects. Graban and Swartz [16] describe the eighth type of waste as the loss of human potential, which many would argue is the most significant waste in terms of failure to engage with staff, to invest in them and to show them their value from the organization’s perspective.

LSS is a highly appropriate methodology for quality improvement in the healthcare setting as it not only places a firm focus on the patient, but also on the necessity of staff inclusion from the beginning, generating buy-in at all levels with a cultural shift towards LSS thinking. LSS was embedded through a tiered training programme, where participants were funded by the hospital’s Lean Academy to complete a University College Dublin graduate diploma programme and facilitated with protected study leave as part of an organizational move toward process improvement. In total the programme took six months to complete.

#### Define

A retrospective audit of cardiac RM device usage was carried out for the period January to March 2016 using departmental logbooks, auditing the use of different devices, recording whether patients were attending clinic for scheduled or unscheduled appointments and noting whether patients were being remotely monitored or not. These data, combined with the absence of protocols to guide follow-up scheduling for RM patients and no dedicated Cardiac Physiologist for RM, resulted in the targeting of the following goals for the project:

- Investigate the impact of RM on unscheduled in-hospital attendance.
- Review and improve patient education guidelines on RM.
- Review and improve information for RM patients based on literature.
- Increase the percentage of suitable patients on RM from 20% to 40%.

The purpose of the initial phase was to document current practice and care in order to establish a baseline for comparison purposes.

#### Measure

A survey was issued over 5 days to all patients attending the clinic, with questions designed to ascertain the reason for attendance, waiting times, and distance travelled. Out of a total of 45 patients who completed the survey, 42% were unscheduled and 58% were scheduled patient appointments.

Clinic attendance was also analysed in the month of April 2016 to gain a clear picture of the number of patients requiring appointments in clinic, those that were unscheduled and whether or not they were being remotely monitored. Out of the total number of follow-ups the majority (64%) were patients physically attending the clinic, with only 24% of patients that month on RM. Of the patients who physically attended, we found that 51% of them were unscheduled patient attendances.

Observations of journeys/experiences from perceptive of the patient (one for RM patient and one for non-RM patient) and staff members, along with surveys of patients and staff were used to verify the collected data. Focus groups of staff and patients were carried out in the cardiac out-patient department to gather perspectives on the service and clinic attendance.

#### Analysis

**Figure 2:**
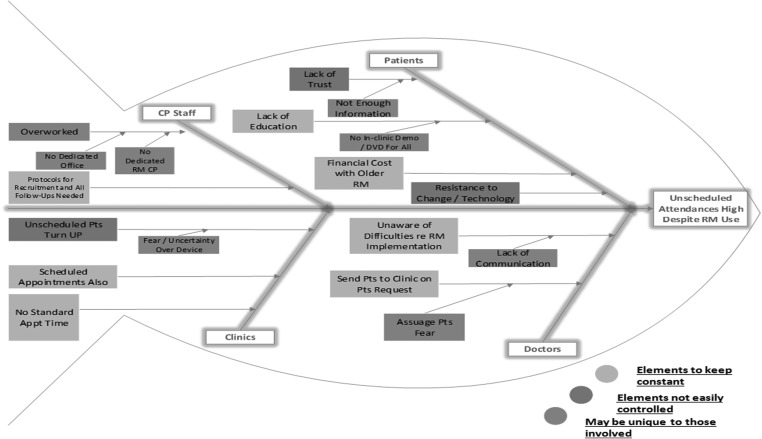
Fishbone analysis of unscheduled attendances to clinic.

An analysis was carried out using a Fishbone diagram, informed by the data collected from staff and patients and an extensive literature review, revealed an opportunity to tackle the internal weaknesses, with success highly possible owing to the buy-in and involvement of stakeholders (see Fig. 2).

We ranked the vital issues to address:

- Lack of guidelines for recruitment and management of patients on RM
- No dedicated cardiac physiologist rostered to RM
- No dedicated office space for RM
- Absence of RM education for patients
- Lack of multidisciplinary awareness of issues surrounding RM recruitment

The information derived from the patient survey revealed that 42% of patients were attending unscheduled and that accounted for most of the time spent on in-clinic appointments as opposed to focusing on the virtual clinic and RM. Many patients struggle with the concept of RM favouring face-to-face communication and opt to attend the device clinic, often unscheduled. This leads to overcrowding of the waiting area, an over-stretching of staff time and a resultant impact on the time allocated to RM review. Staff stated that they required clear guidelines and a dedicated space for RM and referenced the importance of an improved scheduling process on the hospital information system. Patients considered RM a superior service if it was supported with effective education and communication.

#### Improve

Remote monitoring for ICDs is provided free-of-charge in the site of this work and all cardiac devices have RM capabilities in-built, so there is no additional burden to the patient. Additional staff were recruited to the Device Clinic before this project began and therefore, we sought to optimize the work design with these new staff members. The team made physical adjustments to the unit facilitating improved use of space at a cost of €585. An additional staff computer was purchased to facilitate patient enrolment and the remote monitoring of patients. The new layout of the device clinic includes a designated cubicle for RM enrolment, prioritizing and protecting staff time and space to ensure new eligible patients are registered for RM and that RM follow-ups occur as appropriate.

A structured patient education programme was introduced to provide patients with information and resources to inform and reassure them. This programme was designed by the cardiac physiologists with assistance from the project team and employed verbal, written and visual tools based on international guidelines; it also included a patient information manual for each device manufacturer and frequently asked questions. The document is designed to print from the hospital’s electronic patient record information system and patient details populate automatically providing evidence that information was given.

#### Control

At the outset of the control phase, two data points were tracked on a monthly basis to quantify the impact of the project and monitor the sustainability of the changes: remote monitoring activity and unscheduled attendances.

## Results

**Figure 3:**
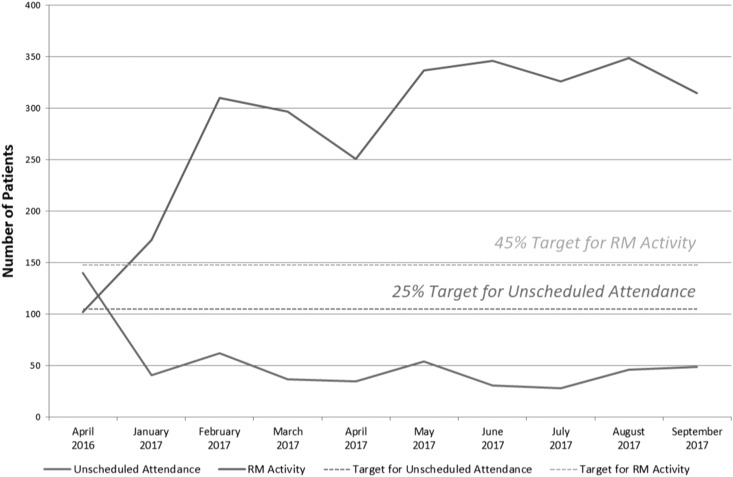
RM activity and unscheduled attendances to clinic

Prior to implementation of the improvements described above, the device clinic was processing 102 RM follow-up checks with 140 unscheduled attendances on average per month. The resultant impact of targeted RM enrolment increased activity to 335 RM follow-up checks, with 41 unscheduled attendances on average per month (see Fig. 3). Control phase results up to October 2017 were compared with baseline data from October 2016. These results demonstrated an increase in remote monitoring activity of 194% (target 45%) with a corresponding decrease in unscheduled attendances of 70% (target 25%). Thus, the project achieved its aims through increasing RM activity and reducing the number of unscheduled patient visits to clinic.

It was estimated that a structured implementation of remote monitoring in line with targets had the potential to yield a time saving of almost 41% as compared to the time spent on equivalent in-hospital follow-up. Sustainability over the control phase, as demonstrated in Fig. 3, was in part a function of confirming RM had the potential to be a long-term solution.

## Discussion

Technological advancements in healthcare, such as remote monitoring of patients with implanted cardiac monitoring devices, has better enabled healthcare teams to effectively and efficiently monitor patients without the requirement for patients to routinely visit clinics. This study employed Lean Six Sigma methods with the objective of designing a process to increase the number of patients availing of cardiac remote monitoring in order to allow for a more detailed monitoring of their heart health, decrease the number of in-person visits to cardiology device clinics, streamline workflow, and release time to educate patients on the benefits of RM in providing safer and higher quality care. Following the implementation of improvement solutions including the re-design of the physical clinic area (to protect time and space for staff to enrol patients in RM) and implementation of a patient education programme linked the hospital IT system, we observed a 70% decrease in unscheduled patients visits to clinic and a concomitant 194% increase in remote monitoring activity. Thus, the aims of this study were achieved and crucially, the new streamlined process better equipped the staff in the device clinic to meet the increased demand for RM from new patients and those requiring regular follow-up.

These findings are particularly pertinent given that previous research has established that RM results in a reduction in the rate of all-cause hospitalizations and in-person clinic visits [4, 6, 7], as well as more timely decision-making when a cardiac event has been observed [3]. Taken together, this evidence suggests that the increase in RM observed has implications for enhanced patient safety and quality of patient care, though this was not directly measured in this study. Staff have also benefited from implementation of the changes through improved workflow and fewer interruptions caused by unscheduled patient visits to clinic.

This work has highlighted the value of LSS approaches in the re-design of workflow and the importance of the involvement of all stakeholders to generate solutions that address identified challenges. For instance, one important approach to data gathering was the patient focus group which provided insight into patients’ perspectives on RM. The focus group provided valuable learning about the importance of patient education to alleviate patients’ fears and anxieties regarding RM and identified the need for feedback from Device Clinic staff after their remote devices had been checked. As part of Phase 2 of this project, a text message service commenced in April 2018 to notify patients that their RM was received and being analysed. A key strength of the study was the collaboration among the project team and cardiac physiologists to develop a patient education programme that informed and reassured patients. As suggested by previous work, such collaborations and social processes are fundamental to quality improvement initiatives [17]. Through involving all stakeholders, the project team successfully developed solutions that met all needs that were then more likely to be implemented and sustained over time.

Another enabler of the success of this project was the team’s ability to link the patient information education intervention to the IT system, so that specific guidance for each patient and each device could be populated by the system immediately and ready for discussion. This could then be monitored for audit purposes to track how often the patient education programme was utilized by clinic staff. This highlights the importance of making change across systems to implement and embed change and to make support staff in sustaining improvement.

The physical changes implemented during the project had, on reflection, a major impact on the success of the project. It created the physical barrier between staff members in the physical clinic and in the RM device clinics. Previously two cardiac physiologists worked in the same area and even though one was assigned to RM, they inevitably were pulled into the physical clinic to deal with the large numbers of unscheduled patients attending. As a result of the space freed up through this project, the creation of an additional ECHO room for the department was facilitated, which had significant knock-on benefits for the in-patient population of the hospital. This additional capacity supported a year-on-year increase of 17% in the number of ECHO tests conducted.

In recommendations for ensuring quality in QI work, it is advocated that teams build capacity and develop a plan for replication and scaling from the beginning [18]. In order to limit the initial scope of this work, this study focused on promoting the use of remote monitoring among patients with implanted cardiac monitoring devices. In the next phase of this work, it is intended that will be adapt and scale up this improvement process to include other remote monitoring devices as we believe we have used a model that is transferable and scalable to other settings where RM is in use. Given the increase in RM observed in this study, it will be important to future work to more closely research impact of remote monitoring on the safety and quality outcomes and on the patient’s experience of care. Another interesting avenue of future research is to interrogate patient’s reasons for unscheduled (unnecessary) visits to clinic when on remote monitoring, and the factors that may be driving inappropriate unscheduled attendances to clinic. Previous research suggests that patient anxiety or lack of trust in RM may be a factor influencing uptake [19] and thus, future work should seek to work closely with patients to co-produce solutions that address such critical issues [20].
