# Corrigendum to: Enhancing efficiency in a cardiac investigations department by increasing remote patient monitoring

**DOI:** 10.1093/intqhc/mzaa002

**Published:** 2020-03-09

**Authors:** Paul Ryan, Caitriona Mcgrath, Iain Lawrie, Caoimhe Fitzsimons, Jack O’Shea, Aoife De Brún

This manuscript has been amended to correct an author’s name. Aoife De Brún was initially incorrectly named in the author list as Jack De Brún. This has now been corrected. Apologies for any confusion caused.

